# Supplementary material for: The Antifungal Activity of Ag/CHI NPs against Rhizoctonia solani Linked with Tomato Plant Health
Source: Plants (Basel). 2021 Oct 25;10(11):2283. doi: 10.3390/plants10112283 (PMC8618975; doi:10.3390/plants10112283)
Supplement: Supplementary file 1 [file plants-10-02283-s001.zip › plants-1368606-supplementary.pdf]

Supplementary information

The Antifungal Activity of Ag/CHI NPs against *Rhizoctonia solani* Linked with Tomato Plant Health

Ameena A. AL-Surhanee[1], Muhammad Afzal[2,3,*], NahlaAlsayedBouqellah[4], Salama A. Ouf[5], Sajid Muhammad[6], Mehmood Jan[6], Sidra Kaleem[7], Mohamed Hashem[8,9], SaadAlamri[8], Arafat Abdel Hamed Abdel Latef[10], Omar M. Ali[11] and Mona H.Soliman[5,12]

1 Biology Department, College of Science, Jouf University, Sakaka 2014, Saudi Arabia; amaserhani@ju.edu.sa
2 Institute of Soil and Water Resources and Environmental Science, College of Environment and Resource Sciences, Zhejiang University, Hangzhou 310058, China
3 Islamic Girls School and College, Parachinar, 26301, KPK, Pakistan
4 Taibah University, Science College, Biology Department, Al Madinah Al Munawwarah 42317- 8599, Saudi Arabia; Nahla.B@hotmail.co.uk
5 Botany and Microbiology Department, Faculty of Science, Cairo University, Giza 12613, Egypt; SaOuFeg@yahoo.com
6 Institute of Crop Science, College of Agriculture and Biotechnology, Zhejiang University, Hangzhou 310058, China; msajid1772@gmail.com (S.M.); mehmoodjan89@gmail.com(M.J.)
7 Ocean College of Science and Engineering, Zhejiang University, 316021, China, kaleemsidra85@yahoo.com
8 Department of Biology , College of Science, King Khalid University, Abha 61413, Saudi Arabia, Drmhashem69@yahoo.com (M.H.); Amri555@yahoo.com (S.A.)
9 Assiut University, Faculty of Science, Botany and Microbiology Department, Assiut 71516, Egypt
10 Botany and Microbiology Department, Faculty of Science, South Valley University, Qena 83523, Egypt; moawad76@gmail.com
11 Department of Chemistry, Turabah University College, Turabah Branch, Taif University, P.O. Box 11099, Taif 21944, Saudi Arabia; om.ali@tu.edu.sa
12 Biology Department, Faculty of Science, Taibah University, Al-Sharm, Yanbu El-Bahr, Yanbu 46429, Saudi Arabia;hmona@sci.cu.edu.eg or monahsh1@gmail.com
* Correspondence: mafzal.kust@gmail.com; Tel.: +92-3348272264

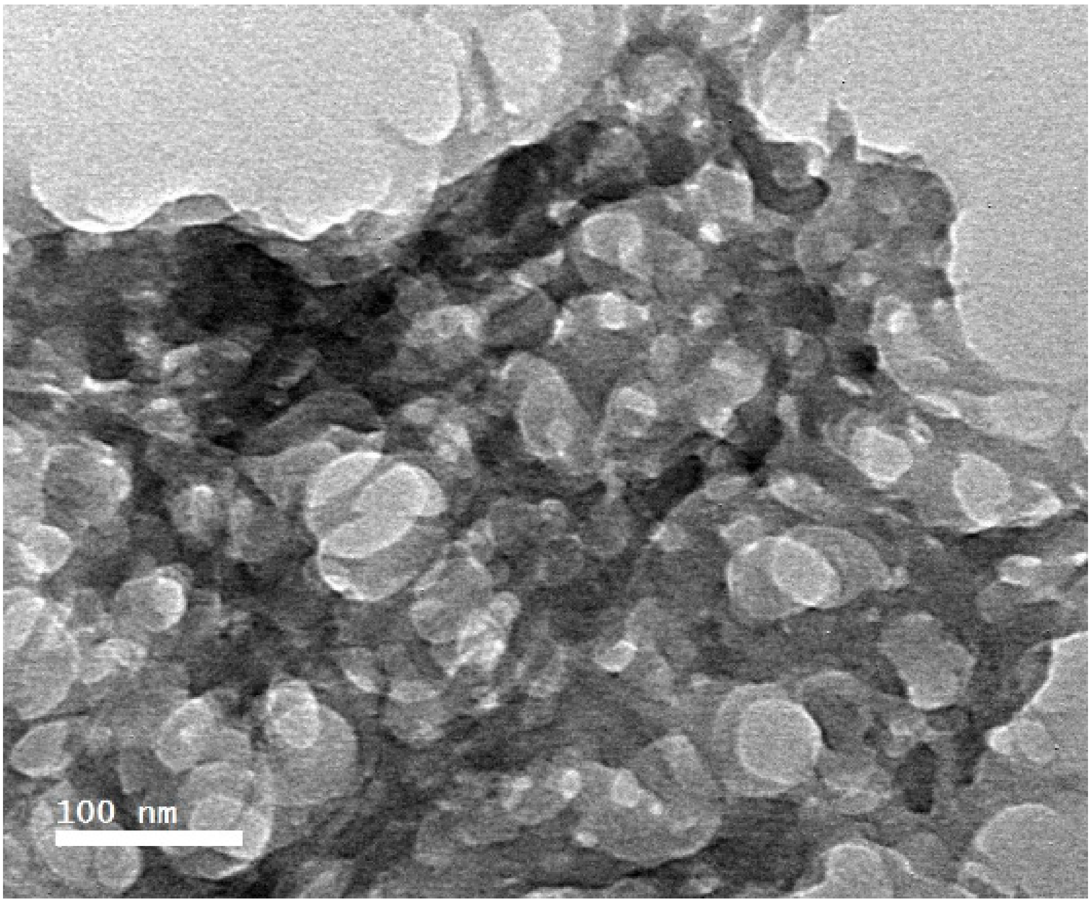

Figure S1. TEM of Silver nanoparticles adhered to CHI NPs in100 nm scale, histogram to average size of the silver NPs and CHI NPs were estimated by imageJ® software.

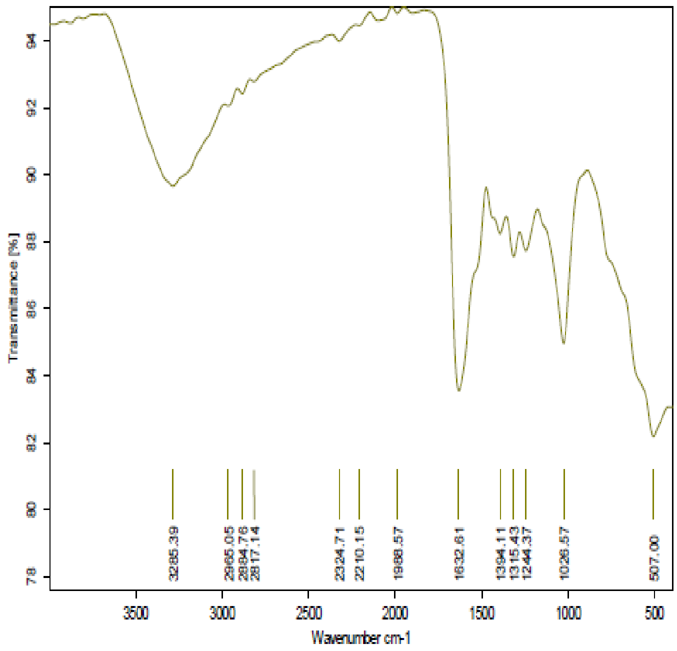

Figure S2(a). FTIR of AgNPs.

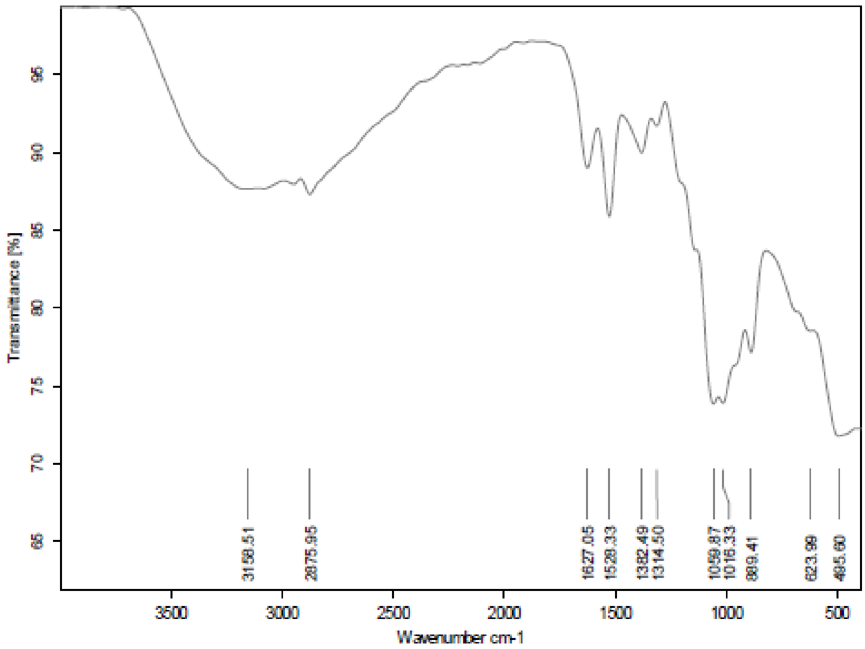

Figure S2(b). FTIR of CHI NPS.

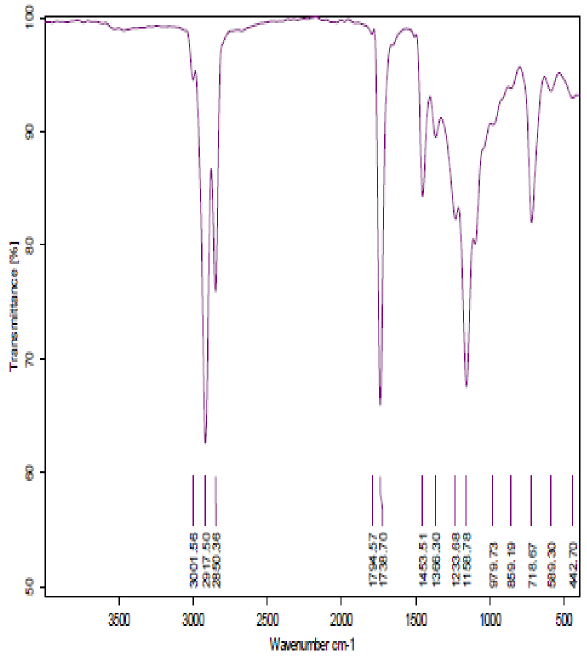

Figure S2(c). FTIR of CHI NPs -AgNPs.

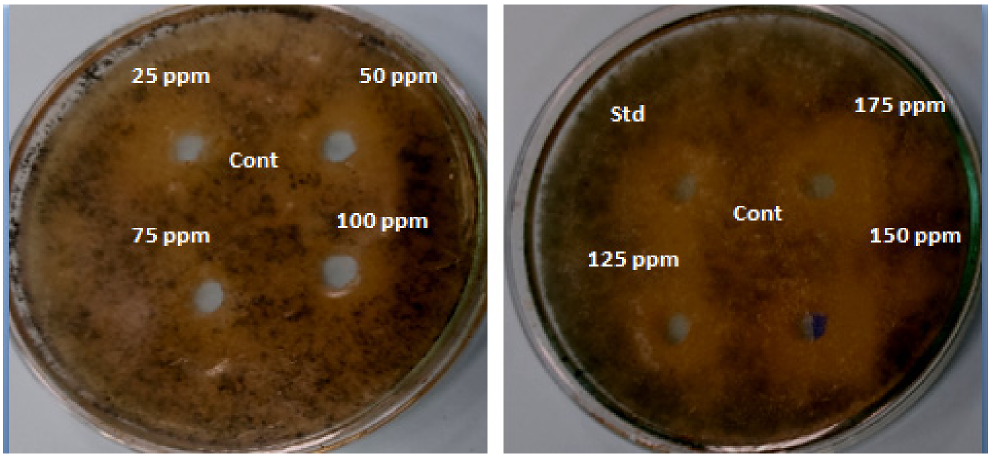

Figure S3. Ag/CHI NC concentrations.

Table S1. Genes and Primers used for Q-PCR.

Gene Name		Sequence
GST	F	5′- ACAAAAAGAGAGCTTGAGCAGC -3’
	R	5′- TCCTGGCAGGCATTTTGTCT -3’
AFPRT	F	5′- GTCGTCTTC TGC CCA TGA TT -3’
	R	5′- ACG TGG AGC ATG GTG TAT CA -3’
PAL1	F	5′- TCG CAA TCG CAA ACA TC -3′
	R	5′- TGC CCT TGA ACC CGT AGT C -3′
Defensin (PR12)	F	5′-CCAAATGCCTCGTCATCT-3′
	R	5′-ATTAGAGTCAAGCTCAAAAGG-3′
Chitenase (PR3)	F	5′-GCGGATCCCAACGCACTGCAACCGATTAT-3′
	R	5′-GCCCATGGAAGGAATCAGTTATGCGCAAAT-3′
β-Actin	F	5′-GTGCCCATTTACGAAGGATA- 3′
	R	5′-GAAGACTCCATGCCGATCAT- 3′
Fungal ITS	F	TCTGTAGGTGAACCTGCGG
	R	TCCTCCGCTTATTGATATGC

Table S2. Concentration (ppm) and inhibitory zones (mm) of Ag-Chitosan and Fungistatin.

**Treatment**	**Concentration (ppm)**	**Growth reduction zone (mm)**
	25	13.483±0.289
	50	17.33±0.288
**Ag-Chitosan concentrations**	75	18.7000±0.05
	100	21.217±0.287
	125	23.93±0.29
	150	25.817±0288
	175	27.117±0.29
**Fungistatin (Std)**	5 µL	24.57±0.29

Table S3. Treatment and germination schedule.

<table>
<tr><th rowspan="2">Soil inoculation with R. Solani 0ne week before transplantation</th><th rowspan="2">Foliar application NC (200 ppm)</th><th colspan="3">Germination</th></tr>
<tr><th>15 days
In peat moss
(15 DAS)</th><th>Transplantation (pot exp.)
7 days
(22 DAYS)</th><th>(Pot exp.)
7 days
(29 DAYS)</th></tr>
<tr><td rowspan="2">Un inoculated</td><td>control</td><td rowspan="4">Seed growing inpeat moss and regularly irrigated with ¼ strength Hoagland solution</td><td colspan="2">regularly irrigated</td></tr>
<tr><td>NC</td><td>NC foliar every 3 days + regularly irrigated</td><td>regularly irrigated</td></tr>
<tr><td rowspan="2">Inoculated</td><td>P</td><td colspan="2">regularly irrigated</td></tr>
<tr><td>P+ NC</td><td>NC foliar every 3 days + regularly irrigated</td><td>regularly irrigated</td></tr>
</table>
